# Effectiveness and safety of prehospital analgesia with nalbuphine and paracetamol versus morphine by paramedics - an observational study

**DOI:** 10.1186/s13049-024-01215-z

**Published:** 2024-05-10

**Authors:** Marvin Deslandes, Martin Deicke, Julia Johanna Grannemann, Jochen Hinkelbein, Annika Hoyer, Matthias Kalmbach, André Kobiella, Bernd Strickmann, Thomas Plappert, Gerrit Jansen

**Affiliations:** 1 University Department of Anesthesiology, Intensive Care Medicine and Emergency Medicine, Johannes Wesling Klinikum Minden, Ruhr University Bochum, Hans-Nolte-Straße 1, 32429 Minden, Germany; 2 Emergency Medical Service, County of Osnabrueck, Am Schölerberg 1, 49082 Osnabrueck, Germany; 3 Department of Anesthesiology and operative Intensive Care Medicine, Hospital of Osnabrueck, Am Finkenhügel 1, 49076 Osnabrueck, Germany; 4 Emergency Medical Services, City and District of Guetersloh, Herzebrocker Strasse 140, 33324 Guetersloh, Germany; 5 Biostatistics and Medical Biometry, Medical School OWL, Bielefeld University, Universitätsstraße 25, 33615 Bielefeld, Germany; 6 Emergency Medical Services, City and District of Fulda, Otfrid-von-Weißenburg-Str. 3, 36043 Fulda, Germany; 7 Emergency Department, Klinikum Fulda, Pacelliallee 4, 36043 Fulda, Germany; 8 Emergency Medical Services of the Order of Malta, Region Hesse, Schmidtstrasse 67, 60326 Frankfurt/Main, Germany; 9 Medical School OWL, Bielefeld University, Universitätsstraße 25, 33615 Bielefeld, Germany

**Keywords:** Prehospital, Pain, Therapy, Emergency

## Abstract

**Background:**

Despite the development of various analgesic concepts, prehospital oligoanalgesia remains very common. The present work examines prehospital analgesia by paramedics using morphine vs. nalbuphine + paracetamol.

**Methods:**

Patients with out-of-hospital-analgesia performed by paramedics from the emergency medical services of the districts of Fulda (morphine) and Gütersloh (nalbuphine + paracetamol) were evaluated with regards to pain intensity at the beginning and the end of prehospital treatment using the Numeric-Rating-Scale for pain (NRS), sex, age, and complications. The primary endpoint was achievement of adequate analgesia, defined as NRS < 4 at hospital handover, depending on the analgesics administered (nalbuphine + paracetamol vs. morphine). Pain intensity before and after receiving analgesia using the NRS, sex, age and complications were also monitored.

**Results:**

A total of 1,808 patients who received out-of-hospital-analgesia were evaluated (nalbuphine + paracetamol: 1,635 (90.4%), NRS-initial: 8.0 ± 1.4, NRS-at-handover: 3.7 ± 2.0; morphine: 173(9.6%), NRS-initial: 8.5 ± 1.1, NRS-at-handover: 5.1 ± 2.0). Factors influencing the difference in NRS were: initial pain intensity on the NRS (regression coefficient (RK): 0.7276, 95%CI: 0.6602–0.7950, *p* < 0.001), therapy with morphine vs. nalbuphine + paracetamol (RK: -1.2594, 95%CI: -1.5770 - -0.9418, *p* < 0.001) and traumatic vs. non-traumatic causes of pain (RK: -0.2952, 95%CI: -0.4879 - -0.1024, *p* = 0.002). Therapy with morphine (*n* = 34 (19.6%)) compared to nalbuphine + paracetamol (*n* = 796 (48.7%)) (odds ratio (OR): 0.274, 95%CI: 0.185–0.405, *p* < 0.001) and the initial NRS score (OR:0.827, 95%CI: 0.771–0.887, *p* < 0.001) reduced the odds of having an NRS < 4 at hospital handover. Complications occurred with morphine in *n* = 10 (5.8%) and with nalbuphine + paracetamol in *n* = 35 (2.1%) cases. Risk factors for complications were analgesia with morphine (OR: 2.690, 95%CI: 1.287–5.621, *p* = 0.008), female sex (OR: 2.024, 95%CI: 1.040–3.937, *p* = 0.0379), as well as age (OR: 1.018, 95%CI: 1.003–1.034, *p* = 0.02).

**Conclusions:**

Compared to morphine, prehospital analgesia with nalbuphine + paracetamol yields favourable effects in terms of analgesic effectiveness and a lower rate of complications and should therefore be considered in future recommendations for prehospital analgesia.

## Background

To improve pain management and reduce the incidence of oligoanalgesia, a wide variety of analgesic concepts for application by paramedics have been evaluated in recent decades [1–8]. Besides non-opioid analgesics, µ-opioid receptor agonists including fentanyl and morphine were investigated. Non-opioid analgesics were shown to be effective in the treatment of mild to moderate pain. Severe pain, which is common in emergency medicine, often requires the use of potent opioids (e.g. fentanyl or morphine). However, therapy with strong opioids yields a complication rate of up to 10%, including potentially life-threatening complications such as, reduced levels of consciousness, as well as respiratory and/or haemodynamic insufficiency [2–8].

The use of ĸ -agonists such as nalbuphine or butorphanol was strongly debated in the 1980s due to high rates of abuse in patients suffering from chronic pain. However, nalbuphine, as a ĸ -agonist and µ-antagonist, has potential advantages when used in acute pain, and its use by paramedics appears reasonable [9–12]: Following a short onset time of 2–3 min, nalbuphine shows good effectiveness for moderate to severe pain. Due to its ceiling effect, the risk of life-threatening adverse effects is low, especially in comparison to pure µ-opioid receptor agonists. In addition, gastrointestinal motility is maintained, leading to lower rates of nausea and vomiting. Nalbuphine causes only little histamine liberation. Nalbuphine showed good effectiveness and safety in various causes of pain, such as trauma, burns and medical causes of pain [10–13]. Despite these advantages, the use of nalbuphine by paramedics is not widespread.

For this reason, the present work examines the use, effectiveness, and complications of prehospital analgesia with nalbuphine + paracetamol in comparison to morphine using data from emergency medical services from the Federal Republic of Germany.

## Methods

The study was approved by the Institutional Review Board of the University of Münster, Germany on February 9, 2022, (file reference 2022-031-fS). Due to its retrospective nature, the requirement of written informed consent was waived by the institutional review board. This article adheres to the applicable Strengthening-the-Reporting-of-Observational-studies-in-Epidemiology (STROBE) guidelines.

### Study design and setting of the study

All emergency operations of the emergency medical services in the Gütersloh district, a district in northwestern Germany, from 01.01.2020–30.06.2022, as well as in the district of Fulda in central Germany, in the period from 01.01.2018–31.05.2023 were included in the investigation. As this is a retrospective data analysis of the Fulda and Gütersloh clusters, the time intervals are not completely identical.

The Gütersloh district’s emergency medical services cover approximately 364,000 inhabitants with 10 ambulance stations, 28 ambulances and 5 emergency physician response vehicles. The population of approximately 223,500 souls in the Fulda district is served by 13 ambulance stations with 27 ambulances, four emergency physicians and one air ambulance. The evaluated data stems from the paramedics’ electronic patient records.

### Selection of participants

All patients ≥ 18 years old with pain requiring therapy, defined as a Numeric-Rating-Scale (NRS) ≥ 4 and analgesic therapy performed by paramedics, following the respective Standard Operating Procedure (SOP) with nalbuphine + paracetamol or morphine were included. Only those patients that consented to the application of analgesics by paramedics were included. Exclusion criteria were patients < 18 years of age, absence of pain requiring therapy, no analgesic therapy performed, prehospital care by a physician, use of analgesics other than nalbuphine + paracetamol or morphine, contraindications to the study drugs or incomplete data.

### Interventions

The medical directors of the emergency medical services of the districts of Gütersloh and Fulda independently created an SOP “Treatment of severe pain requiring therapy.” All paramedics were briefed in the use of their respective SOP. In patients presenting with pain requiring therapy, a structured medical history was taken, regarding past medical history, character, and severity of pain according to the NRS (0 = no pain − 10 = strongest pain). After carrying out basic measures (positioning according to the needs of the patient, insertion of a peripheral venous cannula and administration of a balanced electrolyte solution), basic monitoring (electrocardiogram, oxygen saturation (SpO2) measured by pulse oximetry, non-invasive blood pressure measurement) and oxygen administration as required with a target SpO2 ≥ 94% in patients with a NRS ≥ 4 the analgesic therapy was provided as per local SOP.

### Use of Nalbuphine and Paracetamol in the emergency services of the Gütersloh district

After excluding contraindications to nalbuphine (current opioid-substitution therapy with methadone or recent therapy with µ-agonists, decreased level of consciousness (Glasgow Coma Scale ≤ 14) before the application of analgesics) and paracetamol (e.g. liver disease, allergies), analgesic therapy with these two substances was carried out. In accordance with the SOP the patients received 15 mg/kg body weight of paracetamol intravenously up to a maximum dose of 1,000 mg together with an initial dose of intravenous nalbuphine at a dose of 0.2 mg/kg (patients ≥ 65 years received 0.1 mg/kg nalbuphine i.v.). If this did not provide sufficient analgesia, providers were able to administer a single repeat dose of 0.1 mg/kg (≥ 65 years second and third doses of 0.1 mg/kg nalbuphine) up to a maximum dose of 20 mg of intravenous nalbuphine.

### Use of morphine in the emergency services of the Fulda district

In the Fulda district, morphine was administered after exclusion of contraindications (Glasgow Coma Scale ≤ 14; heart rate < 50/min; respiratory rate < 10/min; systolic blood pressure < 100mmHg and SpO2 < 90% as well as the presence of severe obstructive pulmonary disease). A dose of 0.06 mg/kg body weight was administered. If the pain persisted, repetitive doses of 0.06 mg/kg body weight of morphine were administered every 5–6 min until the specified maximum dose of 10 mg was applied.

### Measurements

The date of the emergency call was recorded along with patient age; sex; cause of pain categorized into traumatic vs. non-traumatic causes of pain [musculoskeletal (non-traumatic pain of the cervical spine, thorax or bones and/or sciatica), visceral (abdominal pain, renal colic), non-traumatic chest pain, headache or other causes of pain], the pain intensity before analgesia and at hospital handover was measured using the NRS, the change in the NRS over the course of treatment (Δ-NRS), the type and dose of the analgesic administered (nalbuphine + paracetamol or morphine). Complications of analgesic therapy such as nausea and/or vomiting, decreased level of consciousness (Glasgow Coma Scale ≤ 14 and/or change in the patient’s mental status), respiratory insufficiency (desaturation, apnea or bradypnea, need for supplemental oxygen administration, assisted and/or controlled ventilation), haemodynamic insufficiency (hypotension with systolic blood pressure < 100 mmHg).

### Outcomes

The primary endpoint was the achievement of adequate analgesia, defined as achieving an NRS < 4, at hospital handover depending on the analgesics administered (nalbuphine + paracetamol vs. morphine). Patients were grouped according to their final score on the NRS for pain in accordance with the German Pain Society (NRS < 4 (no or mild pain); NRS 4–5 (moderate pain); NRS ≥ 6 (severe pain)).

### Analysis

Logistic regression was performed to analyse the primary endpoint. The employed analgesia concept (morphine vs. nalbuphine + paracetamol; “exposure”), as well as age, sex, initial NRS, and cause of pain were included in the model as influencing variables. A linear regression model was used to examine potential associations between exposure and Δ-NRS. This model also included age, sex, initial NRS, and cause of pain as covariates. The secondary end point “occurrence of complications” was analysed via logistic regression as well. Exposure, age, sex, and cause of pain were yet again entered as covariates. Results of logistic regressions were reported using odds ratios (OR), whereas those of linear regression were reported using regression coefficients (RC), each with associated 95% confidence intervals (95%CI) and *p* values. The level of significance was set to *p* ≤ 0.05. All analyses were performed using SAS 9.4 software (SAS Institute Inc., Cary, NC). (SAS Institute Inc., Cary, NC).

## Results

All emergency calls of the emergency medical services in the district of Gütersloh from 01.01.2020–30.06.2022 (*n* = 95,668) and from the district of Fulda from 01.01.2018–31.05.2023 (*n* = 112,655) were included in the evaluation. Analgesic therapy with nalbuphine + paracetamol was carried out in a total of 1,635 patients (1.7% of all emergency cases in Gütersloh; initial NRS:8.0 ± 1.4, median:8.0 (Q1:7.0;Q3:9.0); in Fulda, morphine was used in 173 patients (0.1% of all emergency operations in Fulda; initial NRS:8.5 ± 1.1, median:9.0 (Q1:8.0;Q3:9.0).

Table 1 shows the patient characteristics in the two study groups.

**Table 1:** Characteristics of the patients

Variables	Overall (*n* = 1,808) [*n*(%)]	Nalbuphine + paracetamol (*n* = 1,635) [*n*(%)]	Morphine (*n* = 173) [*n*(%)]
Age in years [mean ± SD]	55.5 ± 21.3	55.1 ± 21.4	59.1 ± 20.3
Female sex	961 (53.1)	877(53.6)	84 (48.5)
Dose of pain medication ( ∑ )			
Nalbuphine in mg [mean ± SD]	8.1 ± 8.4	8.1 ± 8.4	-
Paracetamol in mg [mean ± SD]	990.5 ± 85.	990.5 ± 85.)	-
Morphine in mg [mean ± SD]	4.5 ± 2.3	-	4.5 ± 2.3
Complications			
Nausea and vomiting	41 (2.3)	35 (2.1)	6 (3.5)
Reduction of vigilance	1 (0.1)	0 (0.0)	1 (0.6)
Respiratory insufficiency	2 (0.1)	0 (0.0)	2 (1.2)
Haemodynamic insufficiency	1 (0.1)	0 (0.0)	1 (0.6)
Cause of pain			
Traumatic	759 (42.0)	710 (43.4)	49 (28.3)
Non-traumatic			
Musculosceletal	340 (18.8)	327 (20.0)	13 (7.5)
Visceral	620 (34.3)	553 (33.8)	67 (38.7)
Non-traumatic chest pain	25 (1.4)	0 (0.0)	25 (14.4)
Cephalgia	42 (2.3)	38 (2.3)	4 (2.3)
Others	22 (1.2)	7 (0.4)	15 (8.7)

**Legend** Mg = miligrams; SD = Standard deviation

Table 2 shows the patient characteristics according to the NRS at the end of the operation.

**Table 2:** Patient characteristics grouped according to the Numeric-Rating-Scale at the end of the emergency operations

	NRS at the end < 4			NRS at the end 4–5			NRS at the end ≥ 6		
**Variable**	Overall (*n* = 830) [n(%)]	Nalbuphine + Paracetamol (*n* = 796) [n(%)]	Morphine (*n* = 34) [n(%)]	Overall (*n* = 648) [n(%)]	Nalbuphine + Paracetamol (*n* = 576) [n(%)]	Morphine (*n* = 72) [n(%)]	Overall (*n* = 330) [n(%)]	Nalbuphine + Paracetamol (*n* = 263) [n(%)]	Morphine (*n* = 67) [n(%)]
Age in years [mean ± SD]	55.7 ± 21.3	55.4 ± 21.	59.3 ± 22.7	55.5 ± 22.0	55.1 ± 22.7	59.4 ± 20.2	55.8 ± 20.5	55.1 ± 20.7	58.6 ± 19.4
Female sex	435 (52.4)	414 (52.0)	21 (61.7)	354 (54.6)	320 (55.6)	34 (47.2)	172 (52.1)	143 (54.4)	29 (43.3)
Initial NRS									
Mean ± SD	7.8 ± 1.5	7.8 ± 1.5	7.8 ± 1.2	8.0 ± 1.3	7.9 ± 1.3	8.4 ± 1.1	8.6 ± 1.1	8.5 ± 1.2	9.1 ± 0.9
Median (Q1; Q3)	8.0 (7.0; 9.0)	8.0 (7.0; 9.0)	8.0 (7.0; 8.0)	8.0 (7.0; 9.0)	8.0 (8.0; 10.0)	8.0 (8.0; 9.0)	9.0 (8.0; 10.0)	8.0 (8.0; 10.0)	9.0 (8.0; 10.0)
NRS at the end of the operation									
Mean ± SD	2.1 ± 1.0	2.1 ± 1.0	2.4 ± 0.7	4.4 ± 0.5	4.4 ± 0.5	4.5 ± 0.5	7.1 ± 1.2	7.1 ± 1.2	7.1 ± 1.2
Median (Q1; Q3)	2.0 (2.0; 3.0)	2.0 (2.0; 3.0)	3.0 (2.0; 3.0)	4.0 (4.0; 5.0)	4.0 (4.0; 5.0)	4.0 (4.0; 5.0)	7.0 (6.0; 8.0)	7.0 (6.0; 8.0)	7.0 (6.0; 8.00
NRS reduction									
Mean ± SD	5.7 ± 1.9	5.7 ± 1.9	5.3 ± 1.3	3.6 ± 1.4	3.5 ± 1.4	3.9 ± 1.1	1.5 ± 1.4	1.4 ± 1.4	2.0 ± 1.0
Median (Q1; Q3)	6.0 (4.0; 7.0)	6.0 (4.0; 7.0)	6.0 (5.0; 6.0)	4.0 (3.0; 4.0)	4.0 (3.0; 4.0)	4.0 (3.0; 5.0)	2.0 (0.0; 2.0)	1.0 (0.0; 2.0)	2.0 (1.0; 3.0)
Dose of pain medication in mg (∑)									
Nalbuphine in mg	8.7 ± 8.2	8.7 ± 8.2	-	7.7 ± 8.3	7.7 ± 8.3	-	6.9 ± 9.0	6.9 ± 9.0	-
Paracetamol in mg	991.2 ± 83.3	991.2 ± 83.3	-	988.5 ± 89.4	988.5 ± 89.4	-	992.8 ± 82.8	992.8 ± 82.8	-
Morphine in mg	3.6 ± 2.1	-	3.6 ± 2.1	4.2 ± 1.8	-	4.2 ± 1.8	5.4 ± 2.6	-	5.4 ± 2.6
Complications									
Nausea and vomiting	19 (2.3)	17 (2.1)	2 (5.9)	11 (1.7)	11 (1.9)	0 (0.0)	11 (3.3)	7 (2.7)	4 (6.0)
Reduction of vigilance	0 (0.0)	0 (0.0)	0 (0.0)	0 (0.0)	0 (0.0)	0 (0.0)	1 (0.3)	0 (0.0%)	1 (1.5)
Respiratory insufficiency	1 (0.1)	0 (0.0)	1 (2.9)	0 (0.0)	0 (0.0)	0 (0.0)	1 (0.3)	0 (0.0)	1 (1.5)
Haemodynamic insufficiency	1 (0.1)	0 (0.0)	1 (2.9)	0 (0.0)	0 (0.0)	0 (0.0)	0 (0.0)	0 (0.0)	0 (0.0)
Cause of pain									
Traumatic	345 (41.6)	337 (42.3)	8 (23.5)	276 (42.6)	264 (45.8)	12 (16.7)	138 (41.8)	109 (41.4)	29 (43.3)
Non-traumatic									
Musculosceletal	152 (18.3%)	151 (19.0%)	1 (2.9)	128 (19.7)	123 (21.3)	5 (6.9)	60 (18.2)	53 (20.1)	7 (10.4)
Visceral	303 (36.5)	290 (36.4)	13 (38.2)	210 (32.4)	175 (30.4)	35 (48.6)	107 (32.4)	88 (33.5)	19 (28.4)
Non-traumatic chest pain	9 (1.1)	0 (0.0)	9 (26.5)	11 (1.7)	0 (0.0)	11 (15.3)	5 (1.5)	0 (0.0)	5 (7.5)
Cephalgia	15 (1.8)	15 (1.9)	0 (0.0)	16 (2.5)	13 (2.3)	3 (4.2)	11 (3.3)	10 (3.8)	1 (1.5)
Others	6 (0.7)	3 (0.4)	3 (8.8)	7 (1.1)	1 (0.2)	6 (8.3)	9 (2.7)	3 (1.1)	6 (9.0)

**Legend** Mg = miligrams; NRS = Numeric-Rating-Scale; SD = Standard deviation

At patient handover at the hospital, the mean NRS for therapy with nalbuphine + paracetamol was 3.7 ± 2.0 (median:4.0; Q1:2.0;Q3:5.0; Δ-NRS: mean:4.2 ± 2.3; median:4.0, Q1:3.0;Q3:6.0), when using morphine 5.1 ± 2.0 (median:5.0, Q1:4.0;Q3:6.0; Δ-NRS: mean:3.4 ± 1.7; median:3.0, Q1:2.0;Q3:5.0).

The results of the logistic regression analysis with regards to achieving an NRS < 4 following analgesic therapy are shown in Table 3. Patients with a high initial NRS and patients who received prehospital analgesia with morphine had a lower chance of reaching a score of NRS < 4 after treatment than patients who received nalbuphine in combination with paracetamol (See Table 3).

**Table 3:** Results of logistic regression of the outcome numeric-rating-scale at the end of the operation < 4 vs. ≥ 4

Variables	Odds ratio	95% confidence interval	*p*-value
Age	1,001	0.996–1.006	0.6972
Sex (female vs. male)	0.951	0.783–1.154	0.6083
*Initial Numeric Rating Scale*	***0.827***	***0.771–0.887***	***< 0.0001***
*Morphine vs. Nalbuphine + Paracetamol*	***0.274***	***0.185–0.405***	***< 0.0001***
Traumatic vs. Non-traumatic causes of pain	0.831	0.682–1.014	0.0685

Factors influencing a change in the NRS were the level of the initial NRS, a traumatic vs. non-traumatic cause of pain and therapy with morphine vs. nalbuphine + paracetamol (see Table 4).

**Table 4:** Results of linear regression of the outcome changes of the Numeric Rating Scale

Variables	Regression coefficient	95% confidence interval	*p*-value
Age	0.000249	-0.00424–0.004741	0.9133
Sex (female vs. male)	-0.02899	-0.2176–0.1596	0.7630
*Initial Numeric Rating Scale*	***0.7276***	***0.6602–0.7950***	***< 0.0001***
*Morphine vs. Nalbuphine + Paracetamol*	***-1.2594***	***-1.5770 - -0.9418***	***< 0.0001***
*Traumatic vs. Non-traumatic causes of pain*	***-0.2952***	***-0.4879 - -0.1024***	***0.0027***

Complications of analgesic therapy were observed in a total of 45 patients (2.5%; nalbuphine + paracetamol: *n* = 35 (2.1%) vs. morphine: *n* = 10 (5.8%)) (see Table 1). The logistic regression analysis showed an increased risk of complications following the application of morphine compared to nalbuphine + paracetamol, female vs. male sex and depending on age (see Table 5).

**Table 5:** Results of logistic regression of the outcome complications yes vs. no

Variables	Odds ratio	95% confidence interval	*p*-value
Age	1,018	1,003 − 1,034	0.0200
Sex (female vs. male)	2,024	1,040 − 3,937	0.0379
*Morphine vs. Nalbuphine + Paracetamol*	***2,690***	***1,287–5,621***	***0.0085***
Traumatic vs. Non-traumatic causes of pain	0.806	0.428–1.517	0.5039

## Discussion

The present work evaluates the effectiveness and complications of analgesic therapy by paramedics with nalbuphine + paracetamol in comparison with morphine. After treatment with nalbuphine + paracetamol, patients had lower NRS-scores compared to morphine and a higher chance of achieving an NRS < 4 in the prehospital setting. An NRS < 4 was achieved by 45.9% of patients overall (nalbuphine + paracetamol:48.7% vs. morphine:19.6%). Factors with a beneficial influence on an NRS change were the level of the initial NRS, non-traumatic vs. traumatic cause of pain and therapy with nalbuphine + paracetamol. Overall, complications were rare and predominantly involved nausea and vomiting. Risk factors for complications were therapy with morphine, female sex and the patient’s age.

### Analgesia with opioids in prehospital emergency medicine

Oligoanalgesia continues to represent a relevant problem in prehospital emergency care [14, 15, 16]. The severe pain frequently encountered in prehospital emergency medicine often requires the use of potent opioids. Fentanyl and morphine are commonly used internationally and have been scientifically evaluated in recent years [1–8, 17–19]. In a direct comparison of these substances, comparable analgesic efficacy, but also complications, could be demonstrated for various types of pain [18, 19]. Due to the risk of life-threatening complications following administration of these substances, their use by paramedics is the subject of controversial discussions, whereas the analgesic effectiveness of nalbuphine has been significantly less well studied in this context:

A meta-analysis examining the analgesic effects and safety of morphine vs. nalbuphine showed no differences in analgesic potency between nalbuphine and morphine (pooled-relative-risks:1.01; 95%CI:0.91–1.11; *p* = 0.90), but cannot be fully transferred to the prehospital setting [20]. However, studies from prehospital emergency care indicate that nalbuphine may be particularly suitable for use by paramedics due to its low complication rate [11–13].

To the authors’ knowledge, the present work is the first, comparing prehospital analgesia by paramedics with nalbuphine + paracetamol with morphine and points out, that patients who were treated with nalbuphine + paracetamol, despite comparable initial pain levels, have a significantly higher chance of achieving a pain level of NRS < 4 upon hospital admission. There are various explanations for this: Firstly, while morphine and nalbuphine have almost identical analgesic potencies (morphine:1; nalbuphine:0.7–1.1) and a comparable duration of action of approx. 3–6 h, the time of onset (morphine < 30 min vs. nalbuphine < 3 min) and the time to peak effect (morphine < 90 min vs. nalbuphine approx. 10 min) differ significantly. Hence, a sufficient analgesic effect following the application of morphine may not manifest itself during the prehospital phase of treatment. Considering these pharmacodynamic characteristics, the question arises, whether morphine is suitable for prehospital analgesia, when a rapid onset of the analgesic effect is desired [6, 11, 13, 20].

Secondly, since doses of morphine averaged only 4.5 mg while on average 8.1 mg of nalbuphine were administered, it is likely that an increase in administered doses would have yielded a further improvement of analgesia in both study groups, also yielding the risk of dose-dependent increases in complication rates. It is possible that the ceiling effect of nalbuphine, which offers a high degree of safety, especially regarding respiratory complications and therefore makes titration unnecessary, would be a further advantage for nalbuphine.

Finally, while studies examining the opioid-sparing effect of paracetamol in combination with nalbuphine are lacking to date, it is possible that additive effects could have influenced the results in the study at hand. While additive analgesic effects have been demonstrated for the combination of non-opioid-analgesics with opioids, the data regarding the use of paracetamol remains inconsistent [21, 22]. It would therefore be feasible that a combination of morphine with paracetamol could have caused a further decrease in the rate of patients with oligoanalgesia [23].

### Complications of analgesic therapy

Supplementing studies regarding the effectiveness of various prehospital analgesia concepts by non-medical emergency service personnel, their complications have also been the subject of scientific considerations in the past: While morphine and fentanyl had comparable effects, they also showed similar incidences of complications of up to 18%, including cases of life-threatening adverse effects [6, 18, 19]. The leading complications were respiratory depression (decrease in oxygen saturation: fentanyl ≤ 16.1%, morphine ≤ 4.8%); need for assisted ventilation in few instances with fentanyl at 0.02%). Haemodynamic side effects such as hypotension (fentanyl ≤ 1.5%, Morphine: 0.5%) as well as nausea and vomiting (fentanyl: 1.5%; morphine: 4.8%) were also observed [6]. When comparing the complication rates of analgesia with nalbuphine vs. morphine in the perioperative setting, nalbuphine showed a significantly lower risk of complications for respiratory depression (relative-risk (RR): 0.27 (95%CI: 0.12–0.57; *p* = 0.0007), nausea (RR: 0.78 (95%CI: 0.602–0.997; *p* = 0.048), vomiting (RR: 0.65 (95%CI: 0.50–0.85; *p* = 0.001) and pruritus (RR: 0.17; 95%CI: 0.09–0.34; *p* *<* 0.0001 ) [20]. The results of this study are in line with existing literature and show an efficient pain reduction with low complication rates for prehospital analgesia by paramedics with nalbuphine + paracetamol. While nausea and vomiting were the most common adverse effect in both groups, relevant adverse effects regarding vital functions occurred only after the application of morphine. Therefore, analgesia with nalbuphine + paracetamol may represent a sound alternative, providing effective and safe prehospital analgesia in the hands of emergency medical technicians and paramedics for patients not currently or recently treated with µ-receptor agonists. In this population nalbuphine may cause withdrawal sympoms. Although the administration of µ-receptor agonists is not recommended following nalbuphine, there is the possibility of using µ-receptor agonists when nalbuphine is ineffective or if required otherwise, e.g. in anaesthesia. Due to the nature of competitive antagonism significantly higher doses of µ-receptor agonists may be necessary, requiring careful titration with continuous monitoring. Future studies are needed to evaluate the clinical significance and possible complications of this pharmacological relation.

### Limitations

The limitations of the present work essentially include limitations of retrospective studies: The fact that the patients were not prospectively randomised into one of the two treatment arms, but rather the group comparison was carried out retrospectively, could have led to a distortion of the results and demonstrates the need for prospectively controlled studies. Since it was not possible to examine the onset and time course of prehospital analgesia in a more differentiated manner, it is possible that a further repetition of doses may have led to a further reduction in pain at hospital handover, while also prolonging prehospital on-scene-time and increasing the risk of complications. In both study centres less patients received analgesia as per the evaluated SOPs (1.7% of patients in Gütersloh and 0.1% of patients in Fulda) compared to published rates of severe pain and analgesic administration in other (international) EMS divisions. This may be explained by long-standing legal restrictions on the administration of opioids in the Federal Republic of Germany. Furthermore, as the aim of this work is to compare the analgesic potency and complication rates of nalbuphine + paracetamol vs. morphine, we excluded patients who were treated with any other analgesic substance. Hence, our study does not allow for a statement regarding the incidence of pain requiring therapy in prehospital emergency care in general. Additionally, the baseline characteristics of the patients differ, with more trauma patients and no patients with non-traumatic chest pain being treated with nalbuphine. However, the present results demonstrate the analgesic effectiveness of both analgesic concepts and, for the first time, provide insight into the development of an effective therapy option that may be less prone to complications.

## Conclusions

Prehospital analgesia by paramedics with nalbuphine in combination with paracetamol compared to morphine allows for safe and effective analgesia. Future concepts for prehospital analgesia should therefore implement nalbuphine in combination with paracetamol and evaluate drug interactions during the further course of intrahospital treatment.

## Data Availability

The datasets used and/or analysed during the current study are available from the corresponding author on reasonable request.

## Declarations

### Prior presentations

Parts of the present work have been presented as an abstract at the Congress of the German interdisciplinary association for intensive care and emergency medicine (DIVI 2022) 30.11.-02.12.2022 and DIVI 2023 29.11.-01.12.2023, Hamburg, Germany.

## Abbreviations

95%CI: 95% Confidence Interval
NRS: Numeric-Rating-Scale
OR: Odds ratios
RC: Regression coefficient
RR: Relative risk
SOP: Standard Operating Procedure
